# Disease Control With Delayed Salvage Radiotherapy for Macroscopic Local Recurrence Following Radical Prostatectomy

**DOI:** 10.3389/fonc.2019.00012

**Published:** 2019-02-28

**Authors:** Mohamed Shelan, Seline Odermatt, Beat Bojaxhiu, Daniel P. Nguyen, George N. Thalmann, Daniel M. Aebersold, Alan Dal Pra

**Affiliations:** ^1^Department of Radiation Oncology, Inselspital, Bern University Hospital, University of Bern, Bern, Switzerland; ^2^Department of Urology, Inselspital, Bern University Hospital, University of Bern, Bern, Switzerland; ^3^Department of Radiation Oncology, University of Miami Miller School of Medicine, Miami, FL, United States

**Keywords:** prostate cancer, salvage radiation, local recurrence, macroscopic recurrence, postoperative radiotherapy

## Abstract

**Purpose:** To retrospectively assess clinical outcomes and toxicity profile of prostate cancer patients treated with delayed dose-escalated image-guided salvage radiotherapy (SRT) for macroscopic local recurrence after radical prostatectomy (RP).

**Material and Methods:** We report on a cohort of 69 consecutive patients with local recurrence after RP and no evidence of regional or distant metastasis who were referred for salvage radiotherapy between 2007 and 2016. SRT consisted of 64–66 Gy (2 Gy/fraction) to the prostatic bed followed by dose escalation to 72–74 Gy (2Gy/fraction) to the macroscopic disease. All patients received concurrent short-term androgen deprivation therapy (ADT). Biochemical recurrence-free survival (bRFS) and clinical progression-free-survival (cPFS) were depicted using Kaplan-Meier method. Multivariable Cox proportional hazards regression assessed predictors of survival outcomes. Baseline, acute, and late urinary and gastrointestinal (GI) toxicity rates were reported using CTCAE v4.03.

**Results:** Median time from RP to SRT was 66 months (IQR: 32–124). Median pre-SRT prostate-specific antigen (PSA) was 2.7 ng/ml (IQR: 0.9–6.5). Median follow-up after SRT was 38 months (IQR: 24–66). The 3- and 5-year bRFS were 58 and 44%, respectively. The 3- and 5-year cPFS were 91 and 76%, respectively. Median time from SRT to clinical disease progression was 102 months (IQR 77.5–165). At baseline, 3 patients (4%) had grade 3 urinary symptoms. Six patients (9%) developed acute and six patients (9%) developed late grade 3 urinary toxicity. Five patients (7%) had acute grade 2 GI toxicity. No acute grade 3 GI toxicity was reported. Late grade 3 GI toxicity was reported in one patient (1.5%).

**Conclusions:** Delayed dose-escalated SRT combined with short-course ADT for macroscopic LR after RP was associated with 44% bRFS and 76% cPFS at 5 years. Albeit improved patient stratification is warranted, these data suggest that delayed SRT provides inferior tumor control compared to early intervention.

## Introduction

About 20–40% of all localized prostate cancer patients undergoing radical prostatectomy (RP) develop biochemical recurrence (1, 2). In the absence of overt distant metastasis, salvage radiotherapy (SRT) to the prostatic bed is commonly offered as a potential curative treatment for patients with biochemical or manifested local recurrence (LR).

Several studies have shown that SRT efficacy is highly dependent on the pre-salvage prostate-specific antigen (PSA) level (3–5). However, many urologists including at our institution are reluctant to offer early SRT in the absence of radiologically confirmed local disease. Overall, the two main arguments favoring this approach include lack of conclusive overall survival benefits from prospective clinical trials and a potential increased risk of worsened functional outcomes (6).

The purpose of the current study was to evaluate oncologic outcomes and toxicity rates in a cohort of patients treated with delayed dose-escalated image-guided SRT for macroscopic LR after RP.

## Materials and Methods

### Patient Population

Following approval of institutional and regional ethical review boards, we reviewed charts of 69 consecutive patients who received image-guided SRT after RP due to isolated LR in the prostatic bed between 2007 and 2016 at our Department of Radiation Oncology. Diagnostic imaging included pelvic magnetic resonance imaging (MRI) with or without Choline Positron Emission Tomography/Computed Tomography (PET/CT). Most LR were biopsy proven (41/69 patients, 59%). Baseline patient and disease characteristics as well as baseline (pre-SRT), acute and late toxicities were prospectively collected.

### SRT Protocol

Gross Target Volume (GTV) encompassed the visible macroscopic lesion at the site of recurrence. The GTV delineation on CT simulation was based on available imaging modality that included pelvic MRI with or without Choline PET/CT. The clinical target volume for the entire prostate bed (CTV_prostate_bed) included the prostate/seminal vesicle surgical bed at risk of harboring microscopic disease. The first planning target volume (PTV1) included the CTV_prostate_bed with a margin of 10 mm in all directions except 8 mm posteriorly. The second planning target volume (PTV2) included the GTV with 10 mm-margin in all directions except 8 mm posteriorly.

Radiotherapy was delivered using photon-based intensity modulated radiotherapy (IMRT) with daily cone-beam computed tomography (CBCT) as image-guidance. A dose of 64–66 Gy (2 Gy per fraction) was delivered to PTV1 followed by a dose escalation to 72–74 Gy (2 Gy per fraction) to PTV2. Normal tissue dose constraints as well as bowel and bladder preparations consistently followed institutional guidelines. All patients were treated with concomitant and adjuvant androgen deprivation therapy [luteinizing hormone releasing hormone (LHRH) agonist injections] for 6 months.

### Outcome Measures

During the entire study period, follow-up after SRT was offered at 3, 6, 12, 18, and 24 months and yearly thereafter. Follow up consisted of office visits with review of general health, PSA testing and physical examination including digital rectal examination at suspicion of local progression. For patients who were unable to attend follow up consults, information was retrieved via electronic mail, phone correspondence, and questionnaires sent to primary care physicians and referring urologists.

Biochemical recurrence was defined as post-SRT PSA nadir plus 0.2 ng/ml confirmed by a second PSA rise. Clinical progression was defined as the occurrence of a local, nodal, and/or distant relapse. Diagnostic imaging was performed when clinically indicated and the type of imaging was at the discretion of the treating physician.

Baseline symptoms pre-SRT, acute and late toxicities were tabulated and scored according to CTCAE v4.03 toxicity scale. Late toxicity was defined as toxicity events occurring after 3 months from the end of SRT until the last follow-up date.

### Statistical Analysis

The Kaplan-Meier method was used to depict the probabilities of bRFS and cPFS after SRT. In addition, to account for potential lead-time bias, cPFS was also calculated from the date of RP. Multivariable Cox proportional hazards tested for the effect of pre-SRT PSA level on bRFS and cPFS, adjusting for pathologic stage (pT2-3a/pT3b-4), and GTV (≤ 4.25 cm^3^/> 4.25 cm^3^). Ninety-five percent confidence intervals (CI) were calculated to assess the precision of the obtained estimates. Statistical significance was set at a two-sided p < 0.05 (IBM SPSS Statistics, version 21).

## Results

### Oncologic Outcomes

Patient- and tumor-related characteristics are summarized in Table 1. Median time from RP to biochemical recurrence was 35 months (IQR: 14–64). Median time from RP to SRT was 66 months (IQR: 32–124). Median follow-up time after SRT was 38 months (IQR: 24–66). Median pre-SRT serum PSA was 2.7 ng/ml (IQR: 0.9–6.5).

**Table 1:** Patient and tumor characteristics.

**Variable**	**Value**
Median age at SRT (IQR in years)	71 (66–75)
Median age at RP (IQR in years)	63 (60–66)
Median time to SRT after RP (IQR in months)	66 (32–124)
Median Follow up after SRT (IQR in months)	38 (24–66)
**PREOPERATIVE PSA**	
≤10 ng/ml	28 (40.6%)
>10 ng/ml ≤ 20 ng/ml	11 (15.9%)
>20 ng/ml	10 (14.5%)
Missing	20 (29%)
**T- STAGE**	
pT2-3a	57(82.6%)
pT3b-4	12 (17.4%)
**N-STAGE**	
pN0	58 (84.1%)
pN1	11 (15.9%)
**GLEASON SCORE**^**a**^	
≤6	11 (15.9%)
7	38 (55.1%)
≥8	20 (29%)
**SURGICAL MARGIN**	
Negative	25 (36.2%)
Positive	32 (46.4%)
Missing	12 (17.4%)
**PERINEURAL INVASION**	
Yes	35 (50.7%)
No	16 (23.2%)
Missing	18 (26.1%)
**PRE-SRT PSA**	
0–0.5 ng/ml	10 (14.5%)
>0.5–1 ng/ml	8 (11.6%)
>1–2 ng/ml	9 (13.1%)
>2–4 ng/ml	13 (18.8%)
>4–10 ng/ml	21 (30.4)
Above 10 ng/ml	8 (11.6%)
**HISTOLOGICAL CONFIRMATION**	
Yes	41 (59%)
No	28 (41%)
**GROSS TUMOR VOLUME**	
≤4.25 cm^3^	34 (49%)
>4.25 cm^3^	35 (51%)

a *Gleason score at prostatectomy*.

**Figure 1:**
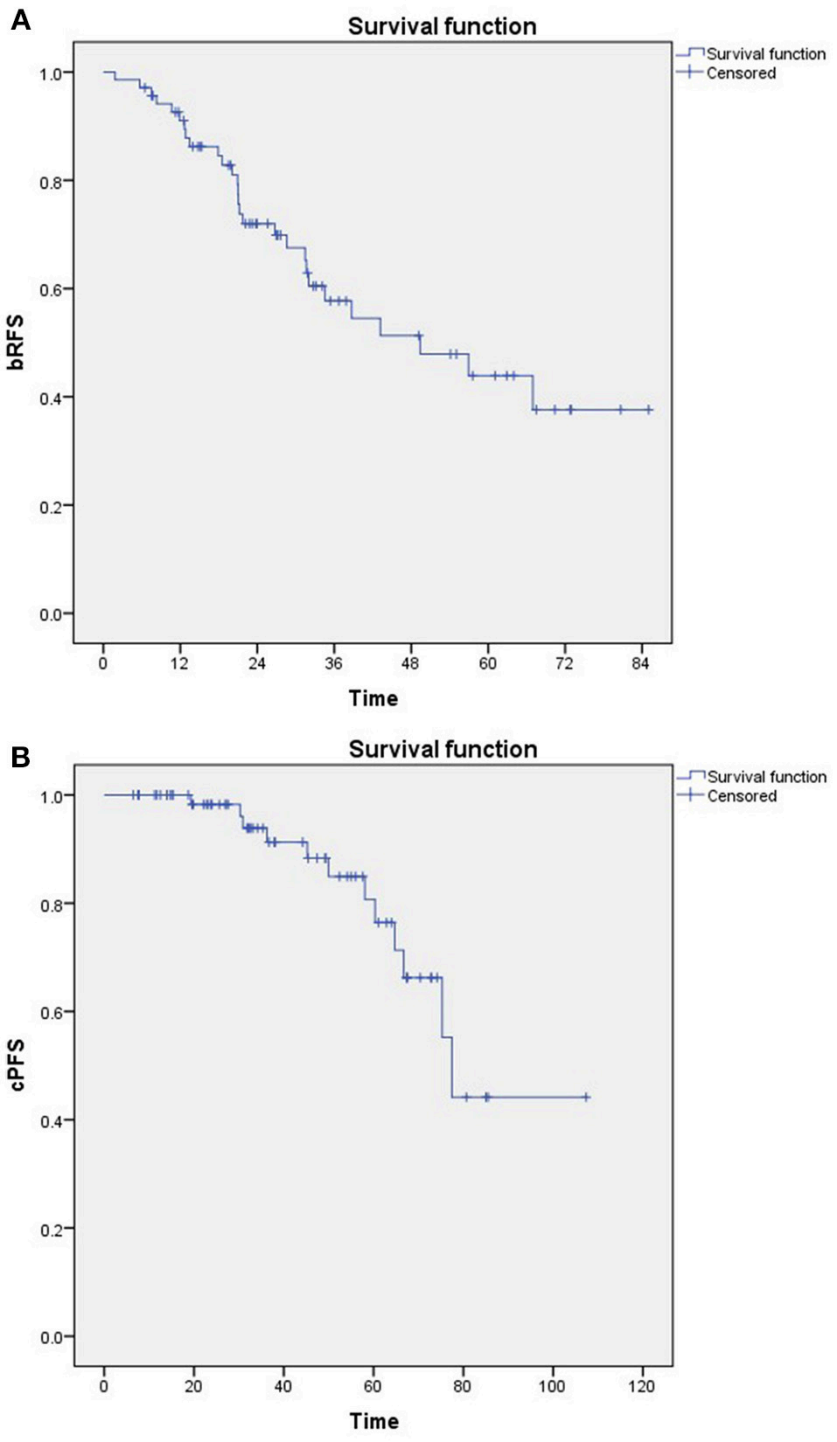
Kaplan-Meier estimates of **(A)** Biochemical relapse-free survival (bRFS) and **(B)** Clinical progression-free survival (cPFS).

A total of 28 patients developed biochemical recurrence after SRT. The 3- and 5- year bRFS were 58 and 44%, respectively (Figure 1A). Furthermore, 12 patients developed clinical progression. The 3- and 5- year cPFS were 91 and 76%, respectively (Figure 1B). In addition, 10- and 15-year cPFS after RP were 90 and 85%, respectively. Median time to clinical progression after RP was 102 months (IQR: 77–156). Only 1 prostate cancer-related death was documented.

In multivariable analysis, pT3b disease and GTV > 4.25cm^3^ were found to be independent predictors of bRFS, but not of cPFS (Table 2).

**Table 2:** Univariable and multivariable logistic regression analysis of factors associated with biochemical recurrence.

	**Univariate**		**Multivariate**	
	**HR (95% CI)**	***p*-value**	**HR (95% CI)**	***p*-value**
**Factors**	**Biochemical recurrence**			
T-stage	3.30 (1.35–8.05)	0.009	3.16 (1.25–8.01)	0.015
Gleason score	2.38 (0.71–7.98)	0.16	–	–
Perineural invasion	2.29 (0.76–6.92)	0.14	–	–
Resection margin	0.62 (0.27–1.43)	0.26	–	–
Pre-RT PSA	3.70 (1.11–12.36)	0.03	2.20 (0.62–7.79)	0.2
Gross tumor volume	4.09 (1.89–8.88)	<0.001	3.79 (1.69–8.47)	0.001

### Toxicity

Prior to SRT, 10 patients (14%) had grade 2, and 3 patients (4%) had grade 3 urinary symptoms, respectively, most frequently urinary retention and incontinence. Following SRT, acute urinary toxicity grade 2 and 3 occurred in 12 (17%) and 6 patients (9%), respectively. Twelve patients (17%) experienced grade 2, and 6 patients (9%) grade 3 late urinary toxicity. The most common symptoms observed after treatment were incontinence and urinary retention. There were no GI symptoms reported prior to SRT. Acute GI toxicity grade 2 was observed in 7 patients (10%), while no grade 3 acute GI toxicity occurred. Late GI toxicity grade 2 was present in 2 patients (3%) and grade 3 in one patient (1.5%). The most frequent symptoms were diarrhea and rectal pain. No grade 4 toxicity was reported at any time (Table 3; Supplementary Material).

**Table 3:** Gastrointestinal and urinary toxicity.

	**Gastrointestinal**			**Urinary**		
	**Baseline**	**Acute (%)**	**Late (%)**	**Baseline (%)**	**Acute (%)**	**Late (%)**
Grade 2	0	7	1.5	12	13	12
Grade 3	0	0	1.5	4	7	9

## Discussion

Our work reports on a cohort of 69 patients homogeneously treated with dose escalated image-guided SRT due to macroscopic LR and a median pre-SRT PSA level of 2.7 ng/ml. At a median follow-up of 38 months, the 3- and 5-year bRFS were 58 and 44%, respectively. The 3- and 5-year cPFS were 91 and 76%, respectively. In this population, pathologic tumor stage and tumor size (GTV) were independent predictors of bRFS, but not of cPFS.

For high-risk prostate cancer patients, LR after RP is a predominant pattern of treatment failure (7). For patients with isolated LR, SRT represents a potentially curative therapeutic option. Indeed, many clinicians favor SRT over upfront adjuvant treatment to allow for postoperative recovery (8) and to avoid possible over-treatment as about 40–50% of the patients can achieve long-term stable disease without further treatment (9, 10). Against this rationale, the optimal timing for SRT and its impact on oncologic outcomes and health-related quality of life is often a matter of debate. Several large observational studies have reported better outcomes when SRT is administrated at a PSA ≤ 0.5 ng/mL (3, 11–14). Consistent with our findings, a large systemic review including 41 retrospective studies reporting on SRT following RP showed that pre-RT PSA is significantly associated with bRFS rates (15). The same work shows an average of 2.6% loss of bRFS for each incremental 0.1 ng/ml PSA increase at the time of SRT. When assessing studies using early salvage as defined by a pre-SRT PSA level of ≤ 0.5 ng/mL, the 5-year bRFS probability is 71% (range: 48–81.8%), which is much higher than the 44% probability reported in the current study (11).

Further, several large studies previously demonstrated the impact of SRT timing on metastasis-free survival. In a retrospective study including 1,106 patients, Stish et al. reported a significant improvement in metastasis-free survival and prostate cancer specific survival when post-RP patients with BCR received SRT at PSA levels ≤ 0.5 ng/mL (16). These findings again argue against prolonged monitoring of detectable post-RP PSA levels that delay initiation of SRT. Some groups have advocated SRT at very early stages, when pre-SRT PSA level ≤ 0.2 ng/mL (17). Although controversy persists, current international guidelines recommend the initiation of SRT at low serum PSA levels (5, 18).

A point of important debate is whether the superior outcomes related to earlier SRT are related to lead time bias (19). Important retrospective series cannot properly rule out this potential bias. Thus, caution should be taken when interpreting most of the results. Nevertheless, a recent analysis presented by Agrawal et al. demonstrated that prostate cancer–specific mortality and all-cause mortality calculated from the time of RP were indeed worse for patients treated with SRT at higher PSA levels (20) These findings corroborated those of Stish et al. (16).

The optimal SRT dose in the setting of macroscopic LR remains unknown. In the absence of macroscopic disease, most guidelines recommend a dose of 64–66 Gy for adjuvant or SRT (5). Retrospective data clearly support dose-escalation to the prostate bed, however the only prospective study assessing dose escalation in the postoperative setting (64 Gy vs. 70 Gy), in the absence of detectable LR, is the SAKK 09/10 trial. This study recently showed low rates of acute grade 2 and 3 GU and GI toxicity with a minor impact in urinary quality of life (21). The ongoing MAPS trial (NCT01411345) randomizes patients with macroscopic recurrence to 68 Gy in 34 fractions vs. 68 Gy in 34 fractions plus boost to MRI-guided macroscopic disease to 76.5 Gy (2.25 Gy per fraction), equivalent to 80 Gy in 2.0 Gy fractions. In our series, 72–74 Gy was used in an unfavorable group of patients with macroscopic LR (mostly biopsy proven) at a median pre-SRT PSA level of 2.7 ng/ml. We found a low toxicity profile in this series with the use of 72-74Gy; however, longer follow-up is required. Although some experiences have shown acceptable toxicity rates with doses up to 80 Gy in the postoperative setting (22), severe GI and GU toxicity may occur with increased RT doses at a longer follow up (23).

Although direct comparisons between studies are challenging, tumor control rates of the present cohort are inferior to prior published series that used SRT at an earlier time-point (Table 4). This is corroborated by results of the phase 3 GETUG-AFU 16 study (29), in which patients with rising post-RP PSA of 0.2–2.0 ng/ml were randomized to SRT vs. SRT plus short term ADT. Patients who received SRT plus short term ADT had a 5-year bRFS of 80% (vs. 44% in our cohort).

**Table 4:** Selected studies using early salvage radiotherapy.

**References**	**No. of patients**	**PSA pre-RT, ng/ml, (range)**	**Follow-up (range)**	**bRFS**
Bernard et al. (24)	69	0.32 (0.1-0.49)	8 yr (0.6–15)	5 yr: 79.8%
Terai et al. (25)	21 of 37	<0.15	31.9 mo (34.3–69.8)	5 yr: 80%
Liauw et al. (26)	34	0.27 (0.05–0.5)	72.4 mo (5.2–136.3)	5 yr: 71%
Briganti et al. (14)	390	<0.5 < 0.3	40.6 mo	2 yr: 92.8% 5 yr: 81.8%
Stephenson et al. (27)	181	0.4 (0.3–0.4)	33 mo (15–132)	3 yr: 69% 5 yr: 61% 6 yr: 48%
Ost et al. (28)	48	0.3 (0.1–0.5)	53 mo (18–132)	5 yr: 77.1%
Current series	69	2.7 ng/ml (IQR: 0.9-6.5)	38 mo (IQR:24-66)	3 yr: 58% 5 yr: 44%

*PSA, prostate-specific antigen; RT, radiotherapy; bRFS, biochemical recurrence-free survival*.

Within limitations of retrospective series along with selection bias, our data suggest that offering early SRT to patients with biochemical relapse at low PSA levels, i.e., before the LR becomes manifest, is essentially preferable. Nevertheless, at low PSA levels, conventional imaging modalities are unable to differentiate loco-regional vs. systemic recurrence. Therefore, prostate bed irradiation may expose patients to unnecessary treatment. One could argue that image-guided delayed SRT could spare many patients from potential SRT-related side effects and quality of life changes. Pre-SRT PSA level was not an independent predictor of cPFS in this cohort. In addition, the vast majority of patients who received delayed SRT survived without clinical progression; albeit at a relatively short follow-up in a group of patients with a long-life expectation (median age at RP was 63 years).

The use of new imaging modalities has been rapidly growing and changing clinical management despite lack of prospective data. Emmet et al. recently showed that PSMA PET is independently predictive of treatment response to radiation. A negative PSMA-PET was a strong predictor of a high response to radiation to the prostate bed (30), also arguing against delayed treatment. In our opinion, delayed SRT should be an option only for unfit patients with limited life expectancy.

The present study has obvious limitations. Although this cohort included a group of patients who were homogeneously treated, it has a relatively small sample. Also, staging procedures were not homogeneously performed (e.g., not all patients had PET-CT scans); thus, some patients may have had regional or systemic disease at the time of SRT. Lastly, 40% of the patients did not have a confirmatory biopsy, hence we relied on the accuracy of multiparametric MRI for the detection and treatment of the LR. Furthermore, a longer follow up is certainly required for more conclusive statements on the impact of delayed SRT in cPFS, distant metastasis and prostate cancer-specific survival.

Taken together, a better stratification of patients who develop biochemical recurrence with or without macroscopic LR after RP is essential for more tailored approaches. Several interesting tools have been studied including patient-specific molecular signatures that could be used to identify the optimal radiotherapy dose (31), radiotherapy timing (32), the efficacy of post-operative radiotherapy (33), and also a promising combination of quantitative multiparametric MRI data with tissue-based gene expression (34).

## Conclusion

Delayed image-guided SRT to macroscopic LR after RP led to 44% bRFS and 76% cPFS at 5 years, suggesting poorer tumor control compared to early SRT. Implementation of novel stratification tools is of utmost importance to advance clinical decision making in the postoperative setting.

## Author Contributions

MS contributed to study conception, clinical analysis, statistical analysis, and manuscript preparation. SO contributed to clinical annotation, clinical analysis, statistical analysis, and manuscript preparation. BB contributed to clinical analysis. DN contributed to clinical analysis and manuscript preparation. GT contributed to manuscript preparation. DA contributed to study conception and manuscript preparation. AD contributed to study conception, clinical analysis and manuscript preparation.

### Conflict of Interest Statement

The authors declare that the research was conducted in the absence of any commercial or financial relationships that could be construed as a potential conflict of interest.
