# Supplementary material for: Disease Control With Delayed Salvage Radiotherapy for Macroscopic Local Recurrence Following Radical Prostatectomy
Source: Front Oncol. 2019 Feb 28;9:12. doi: 10.3389/fonc.2019.00012 (PMC6403145; doi:10.3389/fonc.2019.00012)
Supplement: Supplementary file 1 [file Data_Sheet_1.docx]

Supplementary material

Table S1. Urinary toxicity

|  |  |  |  | Baseline |  | Acute |  | Late |  |
| --- | --- | --- | --- | --- | --- | --- | --- | --- | --- |
| Toxicity |  | Grade |  | % |  | % |  | % |  |
| Dysuria |  | 1 |  | 0 |  | 13 |  | 0 |  |
|  |  | 2 |  | 0 |  | 3 |  | 0 |  |
|  |  | 3 |  | 0 |  | 0 |  | 0 |  |
| Incontinence |  | 1 |  | 12 |  | 10 |  | 10 |  |
|  |  | 2 |  | 10 |  | 9 |  | 12 |  |
|  |  | 3 |  | 0 |  | 0 |  | 0 |  |
| Urinary retention |  | 1 |  | 6 |  | 1.5 |  | 1.5 |  |
|  |  | 2 |  | 0 |  | 0 |  | 0 |  |
|  |  | 3 |  | 4 |  | 6 |  | 7 |  |
| Frequency/Urgency |  | 1 |  | 4 |  | 32 |  | 12 |  |
|  |  | 2 |  | 3 |  | 4 |  | 3 |  |
|  |  | 3 |  | 0 |  | 3 |  | 1.5 |  |
| Hematuria |  | 1 |  | 0 |  | 0 |  | 1.5 |  |
|  |  | 2 |  | 1.5 |  | 1.5 |  | 3 |  |
|  |  | 3 |  | 0 |  | 0 |  | 0 |  |
| Highest Urinary |  | 1 |  | 17 |  | 34 |  | 17 |  |
|  |  | 2 |  | 12 |  | 13 |  | 13 |  |
|  |  | 3 |  | 4 |  | 7 |  | 9 |  |

Table S2. Gastrointestinal toxicity

|  |  |  |  | Baseline |  | Acute |  | Late |  |
| --- | --- | --- | --- | --- | --- | --- | --- | --- | --- |
| Toxicity |  | Grade |  | % |  | % |  | % |  |
| Diarrhea |  | 1 |  | 0 |  | 19 |  | 1.5 |  |
|  |  | 2 |  | 0 |  | 3 |  | 1.5 |  |
| Rectal pain |  | 1 |  | 0 |  | 1.5 |  | 1.5 |  |
|  |  | 2 |  | 0 |  | 6 |  | 0 |  |
| Rectal bleeding |  | 1 |  | 0 |  | 3 |  | 3 |  |
|  |  | 2 |  | 0 |  | 1.5 |  | 0 |  |
|  |  | 3 |  | 0 |  | 0 |  | 1.5 |  |
| Highest GI |  | 1 |  | 0 |  | 19 |  | 4 |  |
|  |  | 2 |  | 0 |  | 7 |  | 1.5 |  |
|  |  | 3 |  | 0 |  | 0 |  | 1.5 |  |

Supplementary figures: Bar charts presenting the frequency of most common gastrointestinal and urinary symptoms at baseline, acute and late follow up

Figure S1

Figure S2

Figure S3

Figure S4

Figure S5

Figure S6

Figure S7

Figure S8
